# Distributions of Hyper-Local Configuration Elements to Characterize, Compare, and Assess Landscape-Level Spatial Patterns

**DOI:** 10.3390/e22040420

**Published:** 2020-04-08

**Authors:** Tarmo K. Remmel

**Affiliations:** Department of Geography, York University, 4700 Keele Street, Toronto, ON M3J 1P3, Canada; remmelt@yorku.ca; Tel.: +1-416-736-2100 (ext. 22496)

**Keywords:** binary, configuration, composition, frequency distribution, variability, spatial pattern, divergence

## Abstract

Even with considerable attention in recent decades, measuring and working with patterns remains a complex task due to the underlying dynamic processes that form these patterns, the influence of scales, and the many further implications stemming from their representation. This work scrutinizes binary classes mapped onto regular grids and counts the relative frequencies of all first-order configuration components and then converts these measurements into empirical probabilities of occurrence for either of the two landscape classes. The approach takes into consideration configuration explicitly and composition implicitly (in a common framework), while the construction of a frequency distribution provides a generic model of landscape structure that can be used to simulate structurally similar landscapes or to compare divergence from other landscapes. The technique is first tested on simulated data to characterize a continuum of landscapes across a range of spatial autocorrelations and relative compositions. Subsequent assessments of boundary prominence are explored, where outcomes are known a priori, to demonstrate the utility of this novel method. For a binary map on a regular grid, there are 32 possible configurations of first-order orthogonal neighbours. The goal is to develop a workflow that permits patterns to be characterized in this way and to offer an approach that identifies how relatively divergent observed patterns are, using the well-known Kullback–Leibler divergence.

## 1. Introduction

Satellites are continually imaging the surface of the Earth and producing representations of real landscapes. These representations have characteristics based on sensor optics and processing decisions that were formally incorporated into the design of the imaging systems. At this stage, the infinite complexity of any imaged landscape is generalized, and subsequent classification of these images into categorical representations even further simplifies the reality of these landscapes through depiction. Such images increasingly offer a principal source of data for vast and complex landscape states, thus enabling nearly limitless investigative opportunities of the environments they characterize.

Through processes of generalization, the finite bounds resulting from a categorical map representation act to greatly improve both the handling of extensive spatial data and analyses based on the data. While any real landscape can be imaged and converted into a gridded categorical map representation in some way, such representations can also result from modelling outputs or secondary analyses of spatial landscape data. The exact characteristics of these categorical maps will be determined by numerous decisions leading to their construction and the limitations of the systems used to acquire and process the data. Regardless, such landscape maps and representations of reality are at the core of many landscape ecological studies (e.g., [1,2,3]).

The measurement and comparison of spatial patterns have received substantial attention in recent decades [4,5,6], with abundant analyses that are directed specifically at the spatial properties of data (e.g., [7]). However, even with hundreds of pattern metrics and approaches [8,9,10,11], no single method has surfaced as a standard for pattern analysis [6]. Much of this speaks to the intrinsic complexity that rasterized spatial patterns encode and depict about landscapes and the processes that generated them [12,13]. With every change to the representation of a given landscape’s state, a new pattern emerges and thus its characterization and quantification will also adapt. Variability introduced by simple changes to characteristics such as image extent, spatial resolution, the number of categorical classes representing a landscape’s state, or the actual configuration of landscapes themselves complicates selecting appropriate metrics and methods for tackling the seemingly simple tasks of pattern characterization and comparison [14,15,16].

In this paper, the idea that by measuring hyper-local configurations (hereafter referred to as pattern elements) across a landscape representation, and recording the frequency distribution of each pattern element, it becomes possible to characterize the broader spatial structure of that landscape via the aggregated relative frequency distribution of the accumulated pattern elements. The pattern elements characterize each possible binary configuration for first-order orthogonal neighbours mapped onto a regular grid of cells. The approach is similar to that implemented by [17] in GeoPAT, in that a histogram of a pattern’s “primitive features” is produced and compared with other histograms using a selection of methods for assessing similarity. Notably different is the ability to design suites of what are deemed to be primitive features in GeoPAT (tending to pick up on topographic niches) and the full specification of all first-order neighbouring pattern cases, as in this paper. The specification of all possible pattern elements (configurational outcomes) provides a consistent base or denominator that describes generic pattern without ties to the data. The collective of all local pattern elements is used to characterize patterns across larger landscapes and to compare them external to any processes that lead to the formation of these patterns. Similarly, this full specification allows for the assessment of how much a landscape deviates from, for example, random, because the underlying baseline pattern elements for specific landscapes can be produced with uncertainty ranges.

At the simplest level, any given cell in a regular grid that is not on an edge will have four nearest orthogonal neighbours. An orthogonal neighbour means that connectivity is determined by having a shared side and are thus not simply joined via a corner. The literature often refers to this type of connectivity as the Rook’s case, following the analogy of chess piece movements [18]. Instances of each possible and unique pattern element are counted at this first-order neighbourhood scale and accumulated across a given landscape to describe the frequency distributions of all pattern elements. This distribution becomes a summary of both composition and configuration at the most local level of a landscape’s representation. A co-occurrence matrix approach [19], commonly implemented in texture analysis to assess categorical co-occurrences of tones/classes between varying locations on a mapped grid, is also a related approach but one that focuses on paired results that would need the production of additional matrices to handle the joint occurrences within a local neighbourhood. The ultimate result would be a similar number of elements but arrived at more laboriously than the elegant solution presented in this paper. While the co-occurrence approach is praised for its rotation invariance, the inability to capture differences in pattern due to rotations would form a limitation and the multiple class case would still lead to a very large number of matrix entries. 

This work has three objectives: (1) to produce characteristic distributions of pattern elements for typical families of landscape patterns through extensive spatial simulation, (2) demonstrate the quantification of divergence between two landscape patterns for assessing similarity and difference, and (3) to implement this method for assessing the prominence of boundaries on a landscape.

## 2. Methods 

**Figure 1:**
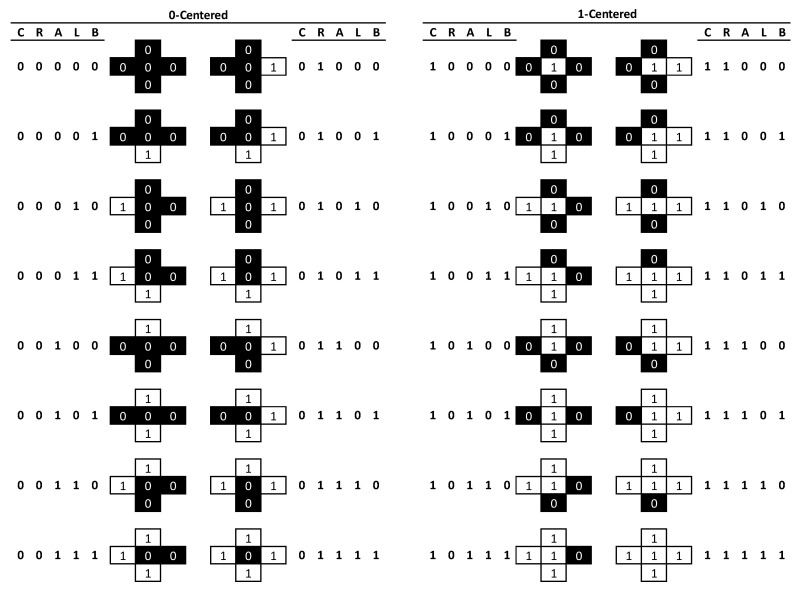
All 32 configurations and encodings for a binary regular grid, considering four orthogonal neighbours. *CRALB* represent Centre, Right, Above, Left, and Below locations, respectively.

**Figure 2:**
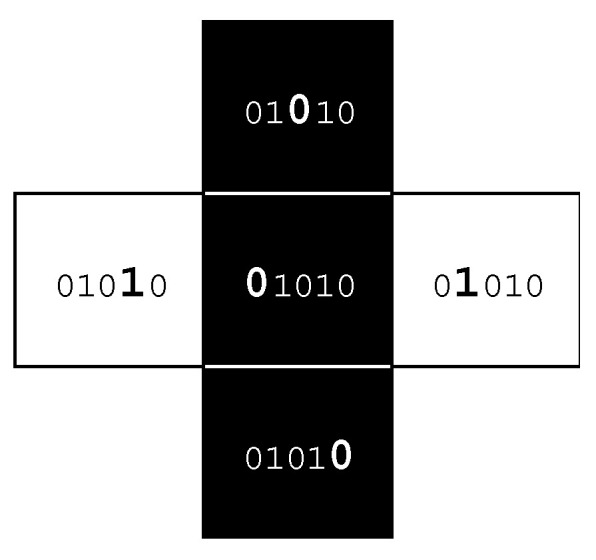
An example of one pattern element (01010), where the binary digit values represent locations *CRALB*, respectively, and are bolded and enlarged to identify the location of black or white cells within a first-order neighbourhood surrounding a cell.

In the binary case, where only two landcover classes are considered, there are 32 possible pattern element configurations (Figure 1). Any focal cell (*C* = centre) can be either black or white (2 states) and the four nearest neighbouring cells (*R* = right, *A* = above, *L* = left, *B* = below) will yield some combination of 16 possible configuration states for each black or white central cell, thus resulting in the total of 2^5^ = 32 possible pattern elements. A single pattern element can be represented by a string of five binary digits (Centre, Right, Above, Left, and Below (*CRALB*)), where 0 indicates a location being black, and 1 being white. Thus, the case 01010 indicates a black cell flanked on the right and left by white cells but black cells above and below (Figure 2). It is possible to count all occurrences of each pattern element and to record these as a frequency distribution. Dividing the frequency for each pattern element by the total frequency of locations assessed provides probabilities of occurrence; the sum of all 32 probabilities will always be 1. 

The proposed approach is presented at the constrained level of first-order orthogonal neighbours and for binary landscapes to reduce an otherwise rapidly increasing complexity resulting from a vast number of possible local configurations. Having fewer pattern elements to track results in dramatically fewer connectivity options to measure and record while maintaining that all connections are equidistant from a focal central cell. Just the additional inclusion of the four first-order diagonal cells would increase the number of combinations to 2^9^ = 512.

Limiting scope to binary representations avoids an escalating number of configurational combinations too large to realistically consider and particularly given that many of these configurations may never actually exist. This constraint reduces the frequency of non-occurrence and thus the overly limiting zero-probability of a pattern element ever occurring, versus having a small probability of occurrence for rare cases. Further, maps with smaller extents (i.e., fewer cells) can be processed, since there is a greater likelihood of sufficient sampling to account for fewer unique pattern elements. For example, a map with only 22 × 22 = 484 cells could never be justified to adequately account for 512 pattern elements (there are too few map cells to even have one occurrence of each pattern element), but may provide sufficient samples for 32 (15 times as many cells as there are possible pattern elements to enumerate). Thus, these constraints permit the processing of images with fewer cells (often akin to having lesser extents) while ensuring that representativeness is preserved, since abundant local measures will statistically characterize more extensive landscape representations. The resulting distribution can be based on a landscape being considered as being on a torus (sides wrap around to form a pseudo-continuous surface) or as a non-torus (whereby the outer rows and columns are omitted from the final tally to avoid edge biases on a non-continuous surface).

**Figure 3:**
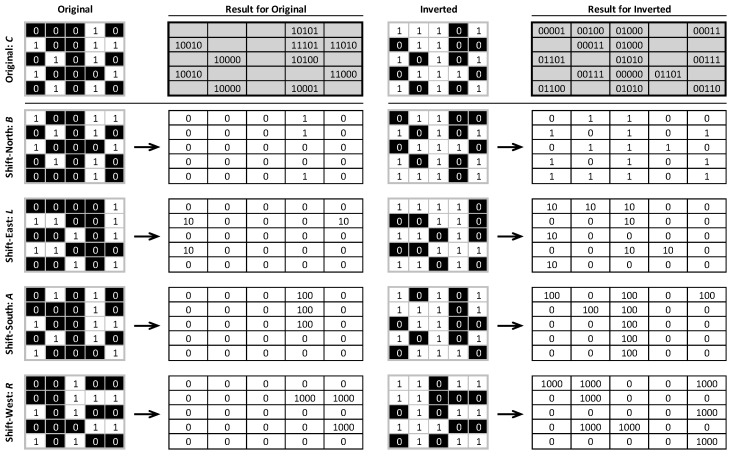
Example of shifting a binary regular grid in the four cardinal directions to produce the *CRALB* set for the original and inverted binary raster maps. Shown are the coding produced and the resulting 5-digit pattern elements for each result (grey) that will be folded together to produce a final result.

Pattern elements are identified by first shifting the input binary map four times (Figure 3)—once in each cardinal direction to produce four new shifted output maps (imagine two playing cards lying on top of each other and the top one being slid relative to the bottom one). Each time one of these shifts occurs, the row or column that is exposed is replicated at the opposite edge; this ensures that the original and shifted layers still align perfectly and have the same extent. The four shifted raster output layers are then multiplied by 1, 10, 100, and 1000 for *R*, *A*, *L*, and *B*, respectively, and further multiplied by the original raster values. These four values are then summed along with 10,000 times the original raster value. The result is an integer that is 5 digits in length, comprising only 0s and/or 1s, each position conforming to the five locations (*CRALB*), indicating the presence of a 0 or 1. This works for locations with 1s in position C; to count occurrences where *C* is 0, the input raster is inverted and the process is repeated. The two independent results are then folded together, and a map of pattern elements is retained.

Once the configurational encoding is complete, the frequency of each unique pattern element is tallied to produce a frequency distribution that is subsequently converted to an empirical probability distribution by dividing each entry by the total number of input cells. In essence, a vector with 32 elements is produced such that each element represents the probability of one unique pattern element occurring in the landscape representation. The ordering of pattern elements is consistent, and thus distributions are directly comparable across any number of assessed landscapes. Implementation was prototyped in Microsoft Excel for testing the logic of this approach and then transmuted into an R [20] function called *patternbits* for operationalization (the R function can readily be downloaded from CRAN (https://cran.r-project.org/package=ShapePattern). The *patternbits* function relies on the *raster* package [21] for drawing functionality but otherwise runs independently.

The first objective is achieved by simulating landscapes along joint-continua of land cover proportion and spatial autocorrelation. Landscapes were simulated using the *CARsimu* function within the *PatternClass* package for R [22]. This simulator provides means for controlling the level of spatial autocorrelation and the proportion of the binary categories while ensuring an underlying stationary and isotropic process to produce the simulated realizations. Composition was varied at 10% intervals from 10% through 90% white-to-black proportion, while spatial autocorrelation was varied from random (RAND) to highly spatially autocorrelated (BUMPY) at 11 intervals to follow the tradition of Remmel and Csillag [14] to produce *N* = (9 compositions) × (11 spatial autocorrelations) × (1000 replicates) = 99,000 images. Images were simulated to have 256 × 256 = 65,536 cells, and pattern elements were enumerated and recorded for each along with the corresponding simulation parameters. Two additional simulations were produced where the landscapes were forced to have strong (CHECK) and intermediate (INTER) degrees of negative spatial autocorrelation, but land cover proportions were maintained as equal (*N* = 2 × 1000 = 2000) in these cases. 

The second objective requires the computation of the Kullback–Leibler (KL) divergence between pairs of empirical probability distributions, stemming from the work by Kullback and Leibler (1951) and widely implemented in various fields [23,24,25,26]. Given that *P* and *Q* are empirical probability distributions defined for a common probability space *X*, then the Kullback–Leibler divergence of *Q* from *P* can be computed by Equation (1) for categorical data. While this relationship is not symmetric or an actual true distance measure, it does identify the relative divergence between two empirical probability distributions [24] and will advise on the structural similarity between two distributions.

(1) $$D_{KL}\left(P∥Q\right)=\underset{x\in Χ}{\sum }P\left(x\right)log\left(\frac{P\left(x\right)}{Q\left(x\right)}\right)$$

The KL divergence is computed among *N* = 1000 randomly selected landscape pairs drawn from the suite of simulated landscapes representing the spectrum of all composition and configuration characteristics and for which pattern elements and their empirical probability distributions are prepared. The paired landscapes are further characterized by the absolute differences between the compositional and configurational parameters in order to identify levels of expected difference. It is hypothesized that the magnitude of the KL divergence corresponds to the structural similarity of the information that two distributions hold, and thus smaller divergences indicate greater similarity.

The third objective is to use the KL divergence to identify the prominence or existence of boundaries where landscape structure changes dramatically. The KL divergence can be computed between or among identified regions, as in the second objective, to identify the degree of difference or similarity between landscape configurations and thus infer the prominence of these defined boundaries. However, this method can serve as a boundary detector as well in order to identify locations of likely configurational boundaries. Presented is a paired moving-window approach that scans across a landscape to quantify changing landscape structures. First, binary landscapes were simulated with broad zones of structural similarity; each image had varying numbers of structural zones and of varying degrees of prominence. Second, two non-overlapping but adjacent 150 × 150 cell subset windows of the simulated image were extracted, their pattern elements were counted, and the KL divergence between them was computed and stored. Third, the windows were shifted by one cell and the process was repeated. When the windows reached the opposite edge of the input image, the processing ended, and the KL divergence was plotted. 

The size of the subset window and the magnitude of its shift between subsequent iterations will affect the outcome. While this is beyond the scope of this paper, the ability to test scaling will permit the adjustment of the application relative to the processes producing the patterns observed (these will vary among studies) and sensitivity to the scale can be assessed. The output plot of the KL divergence provides a quasi-continuous measure of boundary prominence across a landscape (in a specified direction); where the KL divergence is maximized, the boundary is accentuated.

## 3. Results

**Figure 4:**
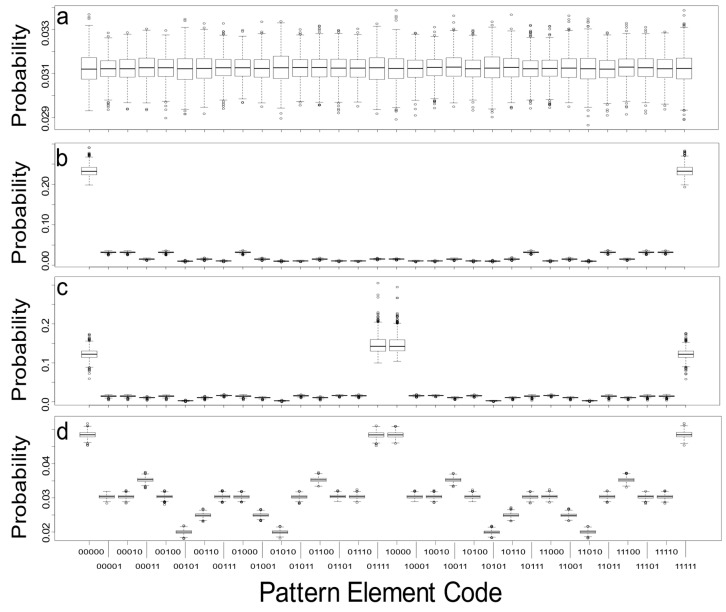
Simulation results for *N* = 1000 replicates for (**a**) RAND, (**b**) BUMPY, (**c**) CHECK, and (**d**) INTER binary landscape patterns with equal class proportions. Shown are the summarized distributions for each of the 32 pattern elements.

The first results depict empirical probability distributions for all pattern elements that represent four extreme cases of simulated binary landscapes with 50% white and 50% black cells. Simulated landscapes, *N* = 1000 each, were produced for random (RAND), highly spatially autocorrelated (BUMPY), intermediately spatially autocorrelated (INTER), and highly checkered or negatively spatially autocorrelated (CHECK) patterns (Figure 4). As expected, the probability of any pattern element is equally likely over multiple simulations for random landscapes (RAND), with the mean for any pattern element converging on $\bar{x_{P}}=\frac{1}{32}=0.3125$. The bounds on this variability are relatively tight (Figure 4a) but do exemplify the expectation that these values will vary due to the random simulation. 

The highly (positively) spatially autocorrelated landscapes (BUMPY) depict high proportions of pattern elements 00000 and 11111, indicating the propensity of like-colours grouping (Figure 4b). The probabilities of these two cases together represent nearly half of the total probabilities. The remaining pattern elements capture the various interfaces and transitions between the classes and happen substantially less frequently. The highly (negatively) spatially autocorrelated landscapes (CHECK) depict high probabilities of pattern elements 00000 and 11111, but also 01111 and 10000, cases where one colour is completely surrounded by the other (Figure 4c). These four pattern elements account for probabilities ranging between 0.1300 and 0.1500, with all other pattern elements being much less probable. The two halves of these distributions are near mirrors of each other (i.e., the first 16 pattern elements have similar distributions to the second set of 16 pattern elements, where differences stem from the inversion of the landscape classes: 0xxxx versus 1xxxx). The intermediate cases (INTER) show a much more dispersed distribution of pattern elements (Figure 4d), but there remain strong similarities with the distributions recorded for CHECK and BUMPY. All of the simulated image replicates are produced by a stationary and isotropic process model.

**Figure 5:**
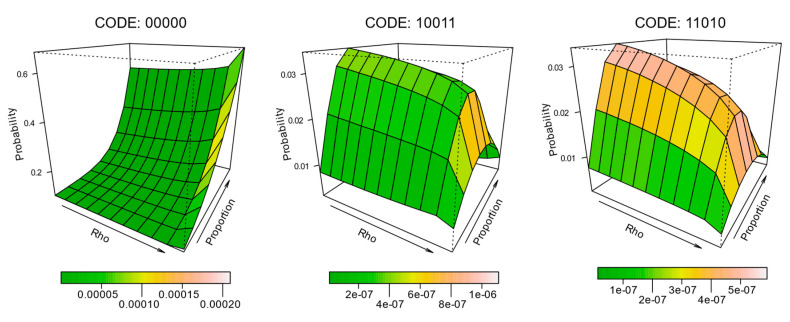
Surfaces demonstrating pattern element probability changes across a range of spatial autocorrelations (Rho ≈ 0.00 to 0.99) and class proportions (10% to 90% white to black). Colouration of the surfaces characterizes the variability of pattern element probability across 1000 simulations.

**Figure 6:**
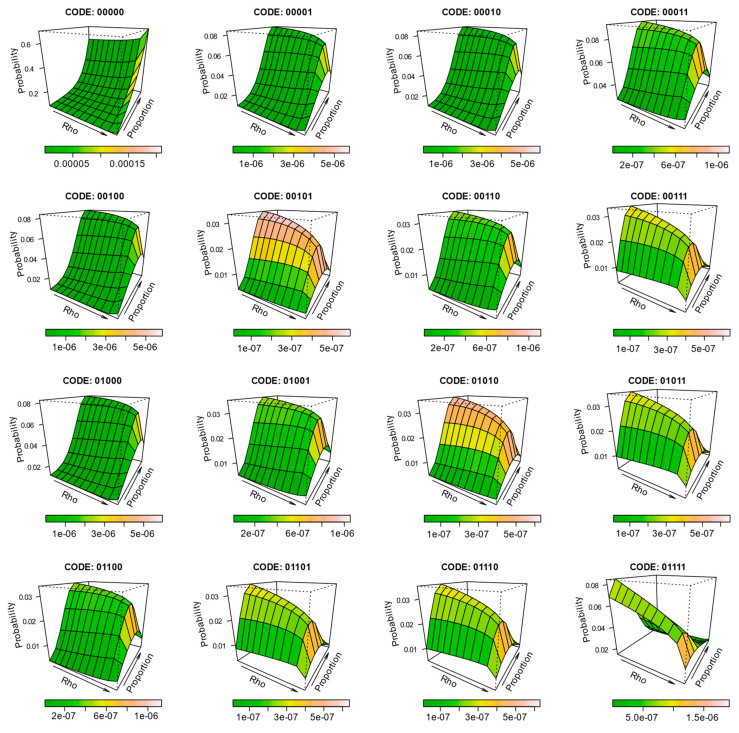
A full half-set of surfaces (others are mirrored cases) demonstrating pattern element probability changes across a range of spatial autocorrelations (Rho ≈ 0.00 to 0.99) and class proportions (10% to 90% white to black). The probability axis is not consistently scaled for illustrative purposes. Colouration of the surfaces characterizes the variability of pattern element probability across 1000 simulations.

To further characterize the effect of composition and configuration on the expectation probability of individual pattern elements, surfaces were constructed for each of the 32 pattern elements, such that the level of spatial autocorrelation and land cover proportion define the margins of the surface, and the surface height represents the mean probability. Figure 5 depicts the results for a selection of these (codes 00000, 10011, and 11010). The surfaces are further coloured to represent the level of variability expressed across the replicates from the simulation. The full half-set (0-centred) surfaces are provided in Figure 6, but they are much smaller than the examples in Figure 5 due to their number.

**Figure 7:**
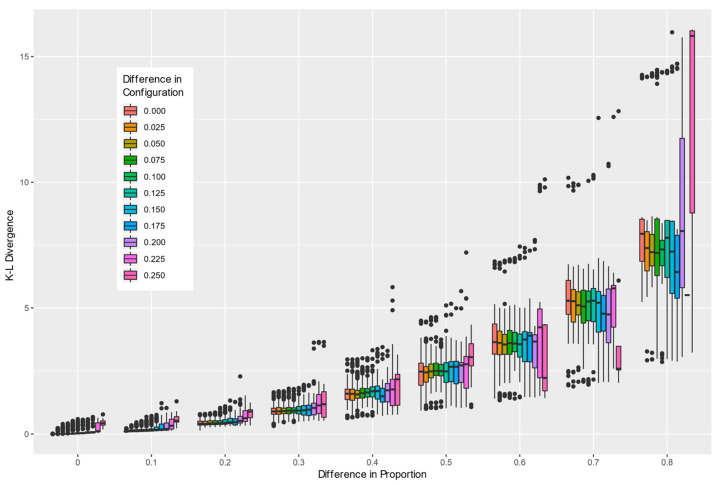
Boxplots depicting the range of variability in Kullback–Leibler (KL) divergence as measured on empirical probability distributions of pattern elements computed for 10,000 randomly selected simulated binary image pairs. The boxplots are grouped by differences in (1) composition and (2) configuration between pairs of compared maps to showcase the ability of the KL divergence to detect landscape structural changes.

Comparing binary landscape patterns based on measurable differences between two distributions of the enumerated pattern elements described in this paper is achieved by computing the KL divergence between the associated paired empirical probability distributions. This approach is well documented and provides a means of establishing baselines and deviations from them. With randomly selected 1000 pairs of maps to compare, the composition and configuration parameters for both maps were retained along with the computed KL divergence between the empirical probability distributions of the pattern elements. Since the absolute composition or configuration are not as informative as the differences between them, additional attributes characterizing the differences in composition and configuration were added to each of the 1000 records. This allowed the summary of the KL divergence relative to both the composition and configuration parameters to be plotted (Figure 7). Differences in composition led to greater differences in the KL divergence than differences in configuration. Greater differences in either composition or configuration led to greater variability in the measured KL diversity between the two landscapes.

**Figure 8:**
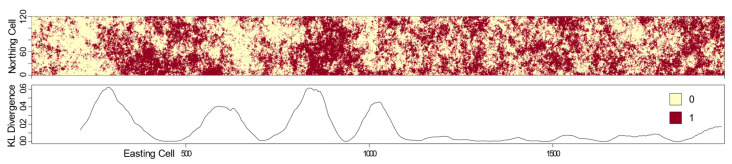
Implementation of the paired moving-window approach to assess the KL divergence across a landscape to identify likely boundary locations. This example used paired 150 × 150 cell, non-overlapping windows.

When a paired moving window was slid across a landscape such that paired subsets of a larger landscape could be iteratively extracted and compared by computing the KL divergence between pattern element empirical probability distributions, the plotted results identified structural changes (i.e., likely boundary locations). The example provided in the top of Figure 8 shows a long east–west trending landscape with two classes (0, 1). Each of the paired moving windows was comprised of non-overlapping but adjacent 150 × 150 cells. The line graph in the bottom of Figure 8 depicts the corresponding computed KL divergence between each of these window pairs as they were slid along the length of this landscape. The curve quantifies and indicates a few important characteristics: (1) the east and west halves of this landscape appear to have differing uniformity, (2) boundaries are transitions as identified by non-abrupt peaks, and (3) the western half of the landscape has several additional boundaries that identify structural differences. The KL divergence curve does not begin at the far left of the landscape due to the window size offset.

## 4. Discussion

It has previously been extensively documented that composition and configuration are the dominant descriptors of a spatial pattern [14,27]; however, while exceptions exist [28], these attributes are generally described by different metrics and in differing units of measurement. The approach introduced here examines binary landscapes and tallies the frequency of each basic pattern element (of which there are 32 in a binary landscape representation). These frequencies are then converted into empirical probability distributions that take on the ability to generalize the spatial structure of that landscape, where its extent and number of cells are not critically important. The distribution itself is telling of the expected pattern observed—both in terms of which land cover class dominates and what types of highly localized spatial arrangements are most likely. 

Code is provided for performing the actions in this paper (and is continually evolving) for use in the open source R environment. This permits the operationalization and modification of functions or their incorporation in popular GIS environments through readily available libraries that interface with R. The current iteration of the computer code has a few limitations that do not affect the proof of concept presented in this paper, but operationally may be problematic: (1) the requirement of an equal number of rows and columns defining input grids, (2) the inability to handle missing data or blanks encoded as NA, (3) data rotations, and (4) currently handling only first-order neighbours. All of these limitations can be considered temporary. 

It is possible to extend the neighbourhood to include the Queen’s case, or even to consider second-order (or beyond) neighbours, but this will dramatically increase the number of possible configurations and also boost the likelihood of highly sparse matrices of pattern element probabilities. Due to the sheer number of combinations, the highly probable existence of ultra-rare cases, and a need for very large assessment landscapes, this additional complexity has been excluded. The handling of grids with unequal numbers of rows and columns should be possible in a near future release of the code. Dealing with missing data (NA) is a bit trickier, but headway is being made on this to accommodate the handling of irregular study areas and data gaps that are common operationally but not theoretically—as in this paper. As for data rotation, this remains an issue that may not actually need a top–down solution. On the one hand, many of the pattern elements can be collapsed with their probabilities aggregated to compensate for reflections; this would mitigate the rotation issue. However, orientation is sometimes important and the current approach provides the precision to detect orientation differences in such cases. Leaving the ability to collapse pattern elements as needed to end users ensures that that decision making is left to those most familiar with their data and needs. What may be interesting in the future, however, is adapting this concept to handle time series by considering the pattern elements in preceding or following timesteps relative to the focal period for assessing landscape change units.

Most land cover products have multiple classes, but the presented approach strictly requires binary representations. While this may be seen as a limitation, it must be emphasized that it is not and that the enumeration for multiple classes or larger numbers of neighbours can be prohibitively tedious and unnecessary [29]. Theoretically, for stationary and isotropic process-generated patterns, even many of the current pattern elements should be reducible, as alluded to by [30], due to uniformity in all directions. This would have the benefit of fewer elements in the empirical probability distributions being compared, a lesser likelihood of null entries, and possibly simpler interpretations, but this would need to be assessed formally and may vary by case. The binary case is further supported by [31], since anomalies in patterns observed locally are more likely to be detected and meaningful than if many more categories were present or measured over larger focal windows.

The slightly more involved calculation of the Jensen–Shannon (JS) divergence, which is symmetric and has a constrained range [32], is also computed and provided as output by the code. However, given the nearly linear relationship between the KL and JS divergences (except for > 70% compositional differences, where the JS divergence saturates), the simpler KL results are accepted as sufficient for the purposes presented here. Operationally, a user can select either (or both) as desired.

Composition is much more readily identified as a change than configuration by human interpretation [30], which is echoed by the KL result in objective 2. Greater changes in composition between two maps lead to substantially larger divergence values, while differences to configuration are subtler. Similarly, larger actual differences in composition lead to greater variability in the KL divergence values, likely a result of a wider array of possible patterns, and thus a greater variation in the distribution of pattern element probabilities is observed. A multiscale analysis may hold interest here, whereby the spatial resolution could be varied across a range of values and the pattern element analysis performed and aggregated akin to a wavelet-type of methodology.

The moving-window scan of the KL divergence values across a landscape provides a unique opportunity to detect structural changes at the most local level, and this characterization is purely data driven. Depending on the size of the moving window and the sliding distance used, the results can be used to detect changes that manifest over varying lag distances and thereby identify boundaries with specific characteristics. The approach identifies locations of potential boundaries and characterizes their strength [33] and their thickness (distance over which they are detectable). This boundary detector could be implemented in multiple directions and then have identified boundaries accumulated into a common boundary layer, with strength and thickness indicated as attributes. This concept has not been tested, but the framework now exists that would facilitate such future investigations.

Related and interesting work in geomorphology and specifically in characterizing topographic units from digital elevation models have resulted in the identification of geomorphons that can be mapped onto a landscape [34,35]. These geomorphons characterize combinations of local slopes and aspects and can classify landscapes into characteristic units that will determining overland flow and erosive effects, but they do rely on the user defined parameters and are not purely pattern-based but related to physical landscape topographic structures. The presented methods in this paper are not intended as an alternative or replacement for such approaches, but methods that may be complimentary in terms of also assessing the spatial pattern of land cover patterns in the same area.

## 5. Conclusions

This study provides a means by which binary landscapes can be summarized by their 32 most primitive pattern elements. These results are converted into probabilities that characterize the dominance of certain elements in describing the overall spatial structure of observed patterns. Extensive simulations identify the impact of changing composition and configuration on the expected distributions of pattern elements, with composition being a greater driver than configuration, and that this aligns with the human interpretive experience as supported by the literature. Furthermore, the use of the KL divergence as a relative measure is useful for comparing the degree of similarity or difference between pairs of landscapes. This approach has the benefit of encoding composition and configuration in a common framework to assess the separation between landscape structures. The use of the KL divergence is also useful for detecting boundaries within landscapes, by detecting structural changes, focusing on changes to the distribution of pattern elements enumerated, rather than direct measures of only a single landscape metric. Likely boundaries as well as their relative prominence (i.e., the KL divergence value) and their abruptness (i.e., distance over which boundaries are detected) are identified.
